# Association of COMT Polymorphisms with Multiple Physical Activity-Related Injuries among University Students in China

**DOI:** 10.3390/ijerph182010828

**Published:** 2021-10-15

**Authors:** Shangmin Chen, Weicong Cai, Shiwei Duan, Lijie Gao, Wenda Yang, Yang Gao, Cunxian Jia, Hongjuan Zhang, Liping Li

**Affiliations:** 1 Injury Prevention Research Center, Shantou University Medical College, Shantou 515041, China; 18smchen@stu.edu.cn (S.C.); 16wccai@alumni.stu.edu.cn (W.C.); 15wdyang@stu.edu.cn (W.Y.); 2 School of Public Health, Shantou University, Shantou 515041, China; 3 Shenzhen Center for Chronic Disease Control, Shenzhen 518020, China; 4 Medical Genetics Center, School of Medicine, Ningbo University, Ningbo 315211, China; duanshiwei@nbu.edu.cn; 5 Department of Epidemiology, Shandong University School of Public Health, Jinan 250012, China; lijiegao@sdu.edu.cn (L.G.); jiacunxian@sdu.edu.cn (C.J.); 6 Department of Sport and Physical Education, Hong Kong Baptist University, Kowloon Tong, Hong Kong 999077, China; gaoyang@hkbu.edu.hk; 7 School of Physical Education, Shanxi Normal University, Linfen 041000, China; swandance@163.com

**Keywords:** Catechol-O-methyltransferase, COMT, polymorphisms, SNPs, physical activity, sports injuries

## Abstract

The catechol-O-methyltransferase (COMT) is a candidate gene to provide promising evidence of psychiatric disorders, but there is a knowledge gap between the genetic factor and multiple physical activity-related injuries (PARIs). The aim of this study was to explore the contribution of COMT to the risk of PARIs among university students in the Chinese Han population. We can further search for the intrinsic risk factors for the occurrence of multiple physical activity injuries and provide a scientific basis for early screening and precise intervention for the high-risk group of college students with multiple PARIs. A 1:1 matched case-control study of 61 PARIs cases and 61 healthy controls were carried out. DNA samples of the participants were isolated from saliva and genotyped on eight SNPs of the COMT gene (rs9265, rs4680, rs6269, rs4818, rs4633, rs165655, rs165656, and rs165722) using the MALDI-TOF MS method. We found that rs6269 and rs4818 were significantly associated with PARIs, and rs6269-GG and rs4818-GG contributed to the reduced risk of PARIs. Further haplotype analysis showed a four-marker C-G-C-G haplotype (rs165722-rs6269-rs4633-rs4818) acted with a protective role in the development of PARIs (*p* = 0.037; OR: 0.474, 95% CI: 0.269 to 0.834). However, the interactions between club membership and rs6269 or rs4818 would significantly increase the risk of PARIs (both *p* < 0.001, OR: 5.121 and 4.977, respectively). This is the first study to find the contribution of COMT to PARIs occurrence, suggesting that the COMT polymorphisms and the gene–environment interactions may alter the risk of PARIs.

## 1. Introduction

Participation in physical activity (PA) is conducive to our individual well-being and helps to achieve and maintain optimal health levels in terms of physical, cognitive and psychological factors [1,2]. In light of these beneficial effects, practically all countries and regions in the world place great emphasis on PA promotion (a public health priority). More positively, there exist several recommendations involving the frequency and duration of PA participation [3,4,5,6]. With such a contemporary focus on a physically active lifestyle, however, a potentially increasing exposure to physical activity-related injury (PARI)—the major adverse consequence of PA participation—can be expected [7,8]. Earlier studies related to the PARI incidence have been primarily confirmed among sports-active populations. For example, basketball, football, and volleyball players in the National Collegiate Athletic Association have injury incidence densities of 6.54–7.97, 8.07–8.44, and 4.69–7.07 per 1000 athlete-exposure, respectively [9,10,11]. Also, our previous study noted that about 22.7% of Chinese university students had at least one PARI episode during the past 12-month period [12]. In fact, PARI could cause substantial medical costs and indirect harmful effects such as PA restriction, psychological fear, and physical discomfort [13,14]. Researchers strongly believe that most PARI could be effectively prevented given the targeted prophylactic interventions, on basis of the better understanding of the injury characteristics and etiology for the specific population [15]. According to previous research, the majority of injuries involved the lower extremities, were acute, newly occurring injuries, and occurred in non-contact situations [9,10,11,16]. Gender and grade differences contributed markedly to the occurrence and severity of PARI [17,18]. In addition, age, body mass index (BMI), PA levels, and family background were also associated with PARI risk [8,17,19]. At present, there is no direct research evidence to clarify the molecular genetic mechanism of PARI. However, since the psychological process involved in injury is mainly related to cognitive flexibility, especially executive function, aggressive behavior, and impulsive risk-taking behavior [17,20,21], by searching for these psychological variables closely related to injury, some genes that may be related to injury will be discovered.

The catechol-O-methyltransferase (COMT) gene is one of the well-studied genes involved in the metabolism of catecholamine neurotransmitters, including dopamine, adrenaline, and noradrenaline [22,23,24,25,26]. The COMT is located at “ 22q11.1–q11.2” and is approximately 27 Kbp in size, with 345 identified polymorphisms [27,28,29]. To date, the most widely studied COMT variant is rs4680 in exon 4, as rs4680 plays a key role in enzymatic activity and cognitive prefrontal cortical function [30]. Owing to the functional G to A polymorphism, rs4680 can result in substitution of valine (Val) to methionine (Met) in codon 108 (in soluble COMT) or 158 (in membrane-bound COMT), generating differences in the ability of the enzymes [24,28]. The enzymatic activity of the Val variant is nearly two to four times higher than the Met variant [30,31]. In addition to Val^108/158^Met, there are other COMT variants associated with the function or expression of regulatory enzymes [32,33]. Furthermore, the important role of COMT haplotype involving several polymorphic sites has also been identified. Notably, the haplotype including four COMT SNPs (rs4818 and rs4680—Val^108/158^Met—in exon 4, rs4633 in exon 3, and rs6269 in P1 promoter) can alter mRNA secondary structure and thereby mediate protein expression levels [34]. Compared to the low-function haplotype ACCG_val_, the high-function haplotype GCGG_val_ expresses an 18- to 25-fold higher enzyme activity, in line with the increased protein levels [34].

Coupled with the previous studies, most genetic association studies of COMT focused on a variety of mental or psychological disorders [35,36], suicide behavior [27], and osteoporotic fracture [37]. No study investigating the association between the COMT and PARIs occurrence was found. Other than the similar influencing non-genetic factors of PARI stated earlier, genetic susceptibility may also play an important part in the occurrence of PARIs—especially in those who experienced PARI repeatedly in the same body part. Therefore, we hypothesized that COMT polymorphisms may alter the risk of PARIs. To test this hypothesis, we explored eight COMT SNPs (i.e., rs9265, rs4680, rs6269, rs4818, rs4633, rs165655, rs165656, and rs165722) through a 1:1 matched case-control study. Consideration of haplotypes are more powerful and effective tools to investigate the effect of genetic variations and gene-environment interaction is becoming increasingly important in the epidemiologic studies [24,38,39], and we also performed the haplotype analysis of COMT polymorphisms, and the interaction of the SNPs within COMT gene and environmental factors. The goal of our study was to find the contribution of COMT to the development of PARIs in the Chinese Han population of general university students.

## 2. Materials and Methods

### 2.1. Participants

**Figure 1:**
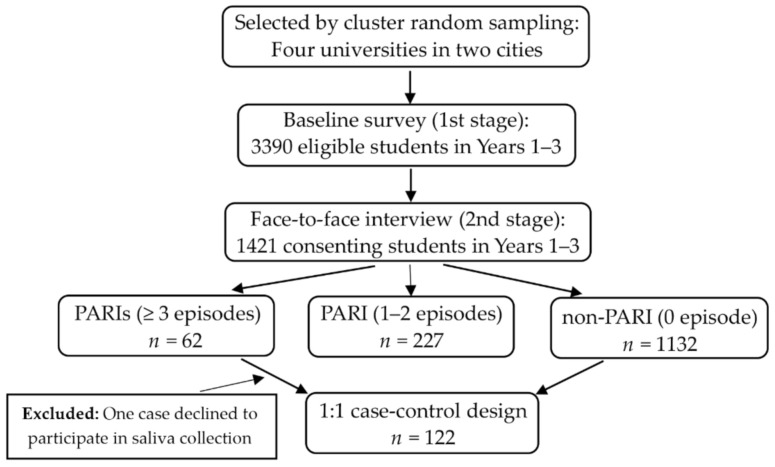
Flow diagram of participants included in the study.

The two-stage design was carried out in four universities in two Chinese cities, namely Shantou and Jinan. Chosen from 3390 students in grades 1 through 3 who participated in the baseline survey during March and April, 1421 students (41.9%) consented to be followed up and completed face-to-face interviews during April and May, 2017. No significant differences in basic demographics (i.e., gender, age, and year level) were found between the subjects in two stages (all *p* > 0.05). The interviewed participants who experienced multiple PARI episodes (at least three injury episodes) in the past 12 months were included in the case group (i.e., PARIs) and those who did not suffer from PARI event during the same period were recruited in the control group (i.e., non-PARI). This was a 1:1 case-control design that matched gender and year level. Given that one participant did not consent to participate in the genetic sub-study (i.e., saliva sample collection), our study finally enrolled 122 individuals identified during a face to face interview as either PARIs cases (*n* = 61, 20.02 ± 1.23 years) and non-PARI controls (*n* = 61, 19.89 ± 1.02 years) (Figure 1).

This study was in accordance with the Declaration of Helsinki, whose protocol was approved by Shantou University Medical College Ethics Committee (SUMC-2016-22). All participants (except for one subject) understood the purpose of our study and completed a written informed consent after our trained personnel verbally explained the study.

### 2.2. Data Collection

The baseline information including gender, age, grade, club membership, screen time (including cellphone and computer) were collected. Moreover, height and weight, and waist and hip circumferences were measured at the same time on the spot, which were calculated as BMI and waist-hip ratio (WHR), respectively.

Adapted from the reliability-validated Chinese version of Children’s Leisure Activities Study Survey (CLASS) [40] and the short version of the Minnesota Leisure Time Physical Activity Questionnaire (MLTAQ) [41], all subjects’ habitual participation in PA were estimated using a series of standardized questions. It has a good reliability (Cronbach’s α = 0.772) in the present study. Participants were asked whether they involved in any PA such as basketball, football, volleyball, and swimming per week in the past 12 months. If yes, they were required to provide information on the frequency and duration of this PA on both a weekday and a weekend, respectively. The weekly PA volume was then calculated.

The term PARI was defined as any injury suffered from during periods of involvement in physical education class, sports activities, or leisure time PA, like sprain, strain, fracture, etc. In addition, a countable PARI episode in this study must occur in the past 12 months and meet at least one of the four judgment criteria, which were fully described in our earlier report [17]. All students were requested to report and count their PARI experiences based on the predefined criteria. In order to ensure that the reported PARI met the aforementioned requirement, those injured participants were asked to provide information about the time of injury occurrence and what types of PA they involved when the injury occurred. Moreover, the injured students were also asked to describe other injury-related details such as causes, types, body parts, places, etc., which contributed to the validation of the PARI measure too. In this study, PARIs cases were defined as those who experienced at least three PARI episodes during the previous 12-month period.

A validated questionnaire was used, and participants were surveyed face-to-face, one-on-one by investigators and had relevant indicators (including height, weight, waist circumference, and hip circumference) measured on-site. In order to reduce bias, improve the degree of cooperation of the survey subjects and ensure the quality of the survey, before filling out the questionnaire, the investigator explained the purpose, significance and precautions of the study, answered relevant questions raised by the students during the filling process, and investigation using recall-promoting interrogation techniques. In order to test the reliability of the data collected by face-to-face interviews, the interviewed information were validated and reliability-tested against the data taken by telephone survey among 50 students with a 1-week interval after their completion of the earlier interviews (average kappa coefficient = 0.775 ± 0.262).

### 2.3. SNP Genotyping

We designed the primers using Assay Design 3.1 software (Sequenom Inc., San Diego, CA, USA), whose details were presented in Supplementary Table S1.

All consenting participants contributed a 2-mL saliva sample using DNA saliva self-collection tube (Beijing Thinkout Sci-Tech Co., Ltd., Beijing, China). DNA was extracted and purified from all saliva samples via a DNA purification kit (Beijing BioTeke Co., Ltd., Beijing, China). DNA fragments were amplified by the polymerase chain reaction (PCR), whose reaction volume was 5 μL containing 20 ng of genomic DNA template. The PCR product was digested with shrimp alkaline phosphatase (Sequenom Inc., San Diego, CA, USA). Following digestion, the amplified fragment was extended in a 9 μL reaction volume and resolved on a 4% agarose gel. All SNPs were genotyped using the high-throughput genotyping platform, conducted by matrix-assisted laser desorption ionization time of flight mass spectrometry (MALDI-TOF MS), and the data was collected via the TYPER 4.0 in the MassARRAY System (Sequenom Inc., San Diego, CA, USA). Furthermore, genotyping was repeated in 5% of all samples to verify the accuracy of the results, revealing that the proportion of concordance was greater than 99%.

### 2.4. Statistical Analysis

Descriptive statistics were used to evaluate the characteristics of the whole subjects. Continuous data that normally distributed were expressed as mean and standard deviation (SD), and the between-group differences were assessed by independent-sample *t* tests. Categorical variables were presented as number and percentage, and tested for differences by Pearson’s *χ^2^* tests.

The power of the sample was calculated by the PS software for power and sample size calculation [42]. The obtained allele and genotype frequencies evaluated using the Hardy–Weinberg equilibrium (HWE) were tested by the *χ^2^* tests for goodness-of-fit. Statistical significance for the between-group differences in allele, genotype and haplotype distribution was calculated by the *χ^2^* tests. Whenever more than one fifth of the cells contained smaller than five subjects, a Fisher’s exact test was used. Odds ratios (ORs) and their corresponding 95% confidence intervals (95% CIs) were generated for associations between allele, genotypes and PARIs. We carried out dominant and recessive models as well, where crude and adjusted association were identified. The linkage disequilibrium (LD) between the SNPs was also calculated and haplotype blocks were identified via the CIs algorithm conducted in Haploview. Statistical analyses were performed with the PLINK 1.07, SPSS 23.0 (SPSS Inc. Chicago, IL, USA), and Haploview 4.0 (Broad Institute, Cambridge, MA, USA). We also applied the generalized multifactor dimensionality reduction (GMDR) using the GMDR 07 to identify the influence of the gene-environment interaction on the risk of PARIs.

All analyses with two-tailed *p*-value less than 0.05 were considered statistically significant.

## 3. Results

There is no difference in gender, club member, age, injury between people who agree to participate in the interview and those who refuse to participate (Table 1). Basic characteristics of the subjects with and without PARIs are presented in Table 2. A total of 60 males and 62 females were included in the present study, respectively. More students were in study year one (*n* = 56, 45.9%). The age of the whole subjects ranged from 18 to 23 years, with a mean age of 20.00 (SD: 1.127). There were no significant differences in the PARIs cases compared to the non-PARI controls in terms of age, BMI, WHR, and screen time (all *p* > 0.05). A significant difference was obtained in club membership between PARIs cases and non-PARI controls (*χ^2^* = 30.500, *p* = 0.000). Also the cases had significantly longer duration in PA participation compared to the controls (1070.41 vs. 781.38 min per week on average, *t* = −2.846, *p* = 0.005).

The success genotyping rate of eight SNPs was between 93.2% and 99.4%. The genotype distribution of all SNPs within the COMT gene were in line with the HWE in the whole study populations (Supplementary Table S2). The frequencies of allele and genotype for eight COMT polymorphisms in cases and controls were summarized in the Table 3.

The genotype distribution of rs6269 differed significantly (*χ^2^* = 6.047, *p* = 0.049). Although rare in both groups, rs6269-GG was significantly less found in cases (5.1% vs. 16.7%; OR: 0.200, 95% CI: 0.049 to 0.818). Regarding the allele frequency, the rs6269-G allele was significantly more common in controls (*χ^2^* = 5.576, *p* = 0.018; 41.7% vs. 27.1%; OR: 0.521, 95% CI: 0.302 to 0.898). Only a moderate difference between cases and controls was observed in the genotype of rs4818 (*χ^2^* = 5.788, *p* = 0.055), rs4818-GG homozygote was more frequent in the control group (14.8% vs. 5.2%; OR: 0.222, 95% CI: 0.054 to 0.923). In addition, more rs4818-G alleles were significantly detected in controls (*χ^2^* = 5.385, *p* = 0.020; 41.0% vs. 26.7%; OR: 0.525, 95% CI: 0.304 to 0.908). There were no significant differences in either genotype or allele frequencies of the remaining COMT polymorphisms between cases and controls.

When applying a dominant or recessive model, we found that the frequency of G allele (i.e., GG + GA) carriers or GG carriers in rs6269 was lower in the case group (dominant model: 49.2% vs. 66.7%; *χ^2^* = 3.745, *p* = 0.053; recessive model: 5.1% vs. 16.7%; *χ^2^* = 4.101, *p* = 0.043). For the dominant model with no adjustments, a reduced risk of PARIs occurrence was found (OR: 0.483, 95% CI: 0.230 to 1.014). This relationship was diminished with the adjustment for gender, grade, and club membership (OR: 0.370; 95% CI: 0.150 to 0.914). After further adjustment in age, BMI, WHR, screen time, and PA participation, the results were further attenuated but remained significant (OR: 0.355; 95% CI: 0.137 to 0.918). There was no significant difference between cases and controls in the recessive model for rs6269 (AA + GA vs. GG). Similarly, the G allele (i.e., GG + GC) carriers or GG carriers of rs4818 in the dominant or recessive model were statistically underrepresented in the cases compared to the healthy control (dominant model: 48.3% vs. 67.2%; *χ^2^* = 4.376, *p* = 0.036; recessive model: 5.2% vs. 14.8%; *χ^2^* = 3.010, *p* = 0.083). A significant association with the lower risk of PARIs was found in a dominant model for rs4818-(GG + GC) vs. CC genotype without covariates (OR: 0.455, 95% CI: 0.217 to 0.956). After controlling various covariates, this association was still significant but weakened gradually. However, no significant difference was obtained in the recessive model for rs4818-(CC + GC) vs. GG genotype. Also, no dominant or recessive models of the remaining SNPs in two groups were found to be significantly correlated (Table 4).

**Figure 2:**
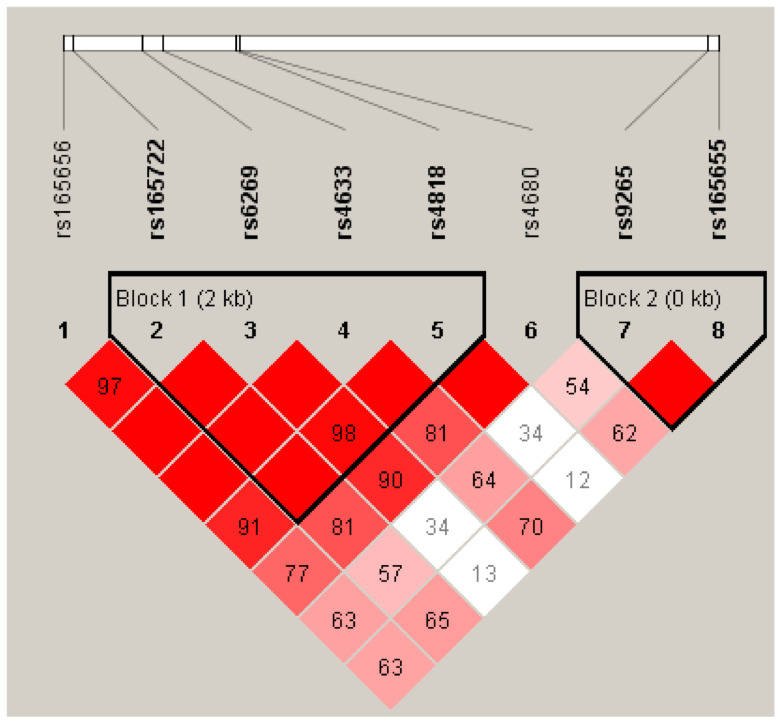
Linkage disequilibrium correlation coefficients of the COMT SNPs.

Employing Haploview to calculate LD, COMT SNPs were found to be in strong LD with each other forming two haplotype modules (Figure 2). The first block was composed of rs165722, rs6269, rs4633, and rs4818, while the second block comprised rs9265 and rs165655. Only the C-G-C-G haplotype in block one was associated with a significantly reduced risk (OR: 0.474; 95% CI: 0.269 to 0.834). The higher frequency of the C-G-C-G haplotype was significantly found in the controls compared to the cases (39.3% vs. 26.8%; *χ^2^* = 4.333, *p* = 0.037). No significant differences regarding the other COMT haplotypes in relation to PARIs could be detected (Table 5).

Investigation into the interaction between COMT SNPs (rs6269 and rs4818) and environmental factors (club membership and PA participation) on the risk of PARIs was performed by the GMDR approach, with gender, grade, age, BMI, WHR, and screen time as covariates. We identified a significantly important interaction model involving club membership and rs6269 based on 1000 times permutation test (cross-validation consistency, CVC: 9/10; test accuracy: 70.7%; *p* < 0.001), and the evidence of this interaction shown by the logistic regression model was 5.121 (95% CI: 1.938 to 13.532). In addition, club membership was also significantly interacted with rs4818 (CVC: 9/10; test accuracy: 71.5%; *p* < 0.001), with an effect of 4.977 (95% CI: 1.881 to 13.171). There were no significant interactions between PA participation and rs6269 or rs4818.

## 4. Discussion

The genetic studies into COMT could provide a promising contribution to the evidence of the pathophysiology, but there is a knowledge gap between the COMT gene and PARI (in particular PARIs occurrence). We conducted an association study involving 61 PARIs cases and 61 matched non-PARI controls. In this study, we found that rs6269 and rs4818 within the COMT gene were independently and significantly associated with PARIs, suggesting that both of them appeared to be involved in the development of PARIs in the Chinese Han population. Regardless of the adjustment for different covariates, the lower risk of PARIs occurrence was observed in the dominant inheritance model for both rs6269 and rs4818, whereas there was no significant association in the recessive model. These results further confirmed that rs6269-GG and rs4818-GG contributed to the reduced risk of PARIs. It is the first time these results of PARIs occurrence and COMT polymorphisms were found, and clearly more research is needed to clarify such an association more broadly.

Some researchers have proposed a “gene-brain-behavior” model, genes cause certain areas of the brain structure and function changes(e.g., hippocampus) [26,29], which affect the individual’s cognition, emotion and behavior [29,43]. Human behavior and the brain network that dominates behavior are formed by the ingenious combination of many genes through development and the environment. Prior research has demonstrated that there is a relationship between between the COMT rs4680 and the impact of COVID-19, which could plausibly be mediated by maladaptive anxiety-related behaviour [44]. Met allele is associated with poorer stress tolerance and the Val allele may confer an advantage in terms of threat perception, leading to better adherence to protective behaviour and minimizing the spread of the disease. The s4818 locus can explain more COMT activity variation, and the G allele at rs4818 locus means that the individual has a higher level of COMT enzyme activity and a lower level of prefrontal dopamine [45]. Studies in the field of cognition have found that subjects carrying the G allele at the rs4818 locus show higher problem-solving ability, but show lower efficiency in planning; The G allele at the rs6269 locus means high levels of COMT enzyme activity and low levels of prefrontal dopamine. Our research may contribute to early identification of individuals at risk in PARI, and suggests a mechanism that can be targeted in future studies aiming to prevent or limit negative outcomes. This may not only help us to reduce the injury during the COVID-19 pandemic, but also provide methods and ideas to explore the biological mechanisms of the impact of COVID-19.

When designing and interpreting association studies of phenotype and genotype, it is necessary to understand the haplotype structures in the human genome because haplotypes are more powerful and effective tools for investigating the potential effect of genetic variations on biological functions and disease susceptibility [38,46]. Many haplotype analysis studies suggested that the SNPs combination led to synergistic effects on enzymatic activity and protein function. [34] Moreover, the distribution of haplotypes varies between various settings and geographic locations and may affect the outcome [47]. In this study, we performed haplotype analysis based on the data for eight COMT polymorphisms generating two haplotype modules. Of these, only the inclusion of the C-G-C-G haplotype in the first block consisting of rs165722, rs6269, rs4633, and rs4818 exerted a significant association with the risk of PARIs (OR: 0.474; 95% CI: 0.269 to 0.834). Our result showed a significantly lower frequency of this haplotype in the PARIs group than the controls, suggesting that it acts as a protective role in the development of PARIs. This is the first study to explore the relationship between the COMT haplotype and PARIs occurrence, and the potential mechanism behind this protective effect of the low activity COMT haplotype on the development of PARIs is still unclear. Further work is needed to validate our findings and to build a more comprehensive view that better reflects the role the COMT haplotypes play in the development of PARIs.

The effects of a single allele might be small, but it might be overwhelmed by other factors [48]. More importantly, complex diseases are more likely to be affected by the gene–gene or gene–environment interactions [49]. For instance, earlier study assumed that the gene–environment interaction might relatively increase the risk for mental disorders with brain injury [48]. In the present study, using the GMDR method to detect the combined contribution of the gene and environment to PARIs occurrence, we found that the significant interactions between club membership and rs6269 or rs4818 played an important role in the development of PARIs. The results of the main effect of rs6269 and rs4818 showed that they were significantly associated with a lower risk of PARIs. Strikingly, when we considered the interactions of these two COMT SNPs with club membership, our results showed a dramatically increased risk of PARIs (OR: 5.121 and 4.977, respectively). The present findings provide a novel insight to the field of gene × environment that the significant joint contribution of club membership and rs6269 or rs4818 may be more responsible for the increased susceptibility of PARIs, helping to further note that the individual contribution of a specific polymorphism might be minor, but the gene–environment interactive effect could be rather significant. Moreover, we did not find significant interaction between PA participation and rs6269 or rs4818 on the PARIs occurrence although the main effects of COMT polymorphisms and PA levels on PARIs occurrence were found in the present study or previous reports [17]. Clearly, these findings warrant replication, and future research should take the gene–gene interaction into consideration as well.

This is the first study to explore the contribution of COMT to the risk of PARIs. But several limitations must be discussed when interpreting the present findings. First, the sample size was small, which may skew results and significance due to the lower statistical power. Thus, our study should be considered preliminary and hypothesis-generating. By contrast with the cross-sectional study design, we carried out a matched case-control study, whose cases and controls came from the same population. This enables us to better preclude the selection bias and obtain specific cause-effect relationship in the risk analysis. However, the participants in the present study were not randomly selected from the baseline survey. This may affect the generalizability of our findings somewhat. Therefore, our results need replication in future research, and further validation in larger sample sizes is undoubtedly necessary to confirm the genetic effect sizes and draw a firm conclusion from a good representative population. Second, although eight polymorphisms were included, we only analyzed the COMT gene in this study. We found that the gene–environment interaction could largely increase the risk of PARIs, but no gene–gene interaction was performed. Previous study noted that, if epistasis exists, the effect of one SNP may be affected by that of another SNP and thereby may impact the power to explore genetic association [50]. Thus, we should take the gene–gene interaction into account in future studies. Third, the information of PA participation was self-reported. The results of 1-week test–retest were validated to be reliable, but we could not fully preclude the possibility of the reporting bias. Hence, the PA measure may bias our gene-environment analyses. Finally, all participants were Han Chinese in order to control the effects of population stratification, but this means that it is not clear the findings can be applied to other races. Further validation in larger samples from other ethnic populations is thereby necessary in order to establish any firm conclusion.

## 5. Conclusions

In summary, this is the first study to find the association of COMT gene with the risk of PARIs. Our results noted that rs6269 and rs4818 were significantly associated with a lower risk of PARIs, especially the rs6269-GG and rs4818-GG. The haplotype of rs165722-C, rs6269-G, rs4633-C, and rs4818-G acted as a protective role in the development of PARIs. Moreover, the significant interactions of club membership with rs6269 or rs4818 could markedly increase the risk of PARIs.

## Figures and Tables

**Table 1:** The differences between participants who agreed to participate in the interview and those who refused to participate.

Characteristics	Responders (*n* = 1421)	Non-Responders (*n* = 1969)	*χ* *^2^* */t*	*p*-Value
Gender			0.938	0.333
Male	377 (26.5%)	552 (28.0)		
Female	1 044 (73.5%)	1417 (72.0)		
Grade			30.89	0.001
Year 1	556 (39.1)	620 (31.5)		
Year 2	447 (31.5)	605 (30.7)		
Year 3	418 (29.4)	744 (37.8)		
Club member			3.111	0.078
Yes	1065 (74.95)	1527 (77.56)		
No	356 (25.05)	442 (22.44)		
Age (Years)	20.09 ± 1.15	20.04 ± 1.38	0.575	0.561
PA participation (min/wk)	691.21 ± 632.64	537.90 ± 702.472	4.382	0.001
PARI			5.340	0.069
0	1 132 (79.7)	1517 (77.1)		
1–2	227 (16.0)	334 (17.0)		
≥3	62 (4.4)	118 (5.9)		

**Table 2:** Characteristics of physical activity-related injuries (PARIs) cases and non-PARI controls.

Characteristics	Controls (*n* = 61)	Cases (*n* = 61)	*χ* *^2^/vs*	*p*-Value
Gender			0.000	1.000
Male	30 (49.2)	30 (49.2)		
Female	31 (50.8)	31 (50.8)		
Grade			0.000	1.000
Year 1	28 (45.9)	28 (45.9)		
Year 2	20 (32.8)	20 (32.8)		
Year 3	13 (21.3)	13 (21.3)		
Club member			30.500	0.001
Yes	10 (16.4)	40 (65.6)		
No	51 (83.6)	21 (34.4)		
Age (Years)	19.89 ± 1.02	20.02 ± 1.23	−0.641	0.523
BMI (kg/m^2^)	20.24 ± 2.77	21.12 ± 3.30	−1.593	0.114
WHR	0.78 ± 0.06	0.79 ± 0.06	−0.086	0.932
Screen time (hours/day)	7.16 ± 2.78	6.66 ± 2.81	0.989	0.324
PA participation (min/wk)	781.38 ± 575.87	1070.41 ± 545.52	−2.846	0.005

BMI, body mass index; WHR, waist-hip ratio; PA, physical activity. Figures in parentheses indicate percentages.

**Table 3:** Allele and genotype distributions of the SNPs in the COMT gene in the PARIs cases and the non-PARI controls.

SNPs	Controls	Cases	*χ^2^*, *p*-Value	OR
rs9265			0.482, 0.786	
CC	17 (28.8)	16 (27.6)		1.000 (ref.)
AC	25 (42.4)	28 (48.3)		0.875 (0.327–2.339)
AA	17 (28.8)	14 (24.1)		1.190 (0.499–2.840)
A%	50.0	48.3	0.070, 0.792	0.933 (0.559–1.558)
rs4680			3.607, 0.165	
GG	32 (56.1)	23 (39.7)		1.000 (ref.)
AG	24 (42.1)	32 (55.2)		1.855 (0.874–3.939)
AA	1 (1.8)	3 (5.2)		4.174 (0.408–42.716)
A%	22.8	32.8	2.835, 0.092	1.649 (0.919–2.959)
rs6269			6.047, 0.049	
AA	20 (33.3)	30 (50.8)		1.000 (ref.)
GA	30 (50.0)	26 (44.1)		0.578 (0.267–1.250)
GG	10 (16.7)	3 (5.1)		0.200 (0.049–0.818)
G%	41.7	27.1	5.576, 0.018	0.521 (0.302–0.898)
rs4818			5.788, 0.055	
CC	20 (32.8)	30 (51.7)		1.000 (ref.)
GC	32 (52.5)	25 (43.1)		0.521 (0.241–1.126)
GG	9 (14.8)	3 (5.2)		0.222 (0.054–0.923)
G%	41.0	26.7	5.385, 0.020	0.525 (0.304–0.908)
rs4633			1.995, 0.369	
CC	39 (63.9)	33 (54.1)		1.000 (ref.)
TC	20 (32.8)	23 (37.7)		1.359 (0.637–2.899)
TT	2 (3.3)	5 (8.2)		2.955 (0.538–16.239)
T%	19.7	27.1	1.854, 0.173	1.514 (0.832–2.756)
rs165655			0.081, 0.960	
GG	17 (27.9)	18 (30.0)		1.000 (ref.)
AG	34 (55.7)	32 (53.3)		0.889 (0.291–2.018)
AA	10 (16.4)	10 (16.7)		0.944(0.315–2.834)
A%	44.3	43.3	0.021, 0.884	0.963 (0.579–1.600)
rs165656			1.395, 0.498	
GG	38 (63.3)	32 (53.3)		1.000 (ref.)
CG	19 (31.7)	23 (38.3)		1.438 (0.667–3.099)
CC	3 (5.0)	5 (8.3)		1.979 (0.439–8.929)
C%	20.8	27.5	1.455, 0.228	1.441 (0.795–2.615)
rs165722			1.019, 0.601	
CC	37 (61.7)	32 (55.2)		1.000 (ref.)
TC	21 (35.0)	22 (37.9)		1.211 (0.565–2.957)
TT	2 (3.3)	4 (6.9)		2.312 (0.397–13.469)
T%	20.8	25.9	0.835, 0.361	1.326 (0.723–2.429)

SNPs, single nucleotide polymorphisms; COMT, catechol-O-methyltransferase; A, Adenine; G, Guanine; C, Cytosine; T, Thymine. Figures in parentheses indicate percentages or 95% CIs.

**Table 4:** Association of the dominant model and recessive model in the PARIs cases and the non-PARI controls.

SNPs	OR (95% CI) ^1^	OR (95% CI) ^2^	OR (95% CI) ^3^
rs9265			
DOM	1.062 (0.457–2.378)	0.693 (0.267–1.800)	0.719 (0.273–1.897)
REC	0.786 (0.345–1.792)	0.875 (0.332–2.302)	0.850 (0.297–2.437)
rs4680			
DOM	1.948 (0.928–4.090)	1.684 (0.726–3.908)	1.938 (0.798–4.699)
REC	3.055 (0.308–30.271)	2.166 (0.168–27.946)	0.967 (0.043–21.733)
rs6269			
DOM	0.483 (0.230–1.014)	0.370 (0.150–0.914)	0.355 (0.137–0.918)
REC	0.268 (0.070–1.028)	0.426 (0.096–1.887)	0.351 (0.075–1.641)
rs4818			
DOM	0.455 (0.217–0.956)	0.389 (0.161–0.938)	0.364 (0.144–0.924)
REC	0.315 (0.081–1.229)	0.484 (0.107–2.182)	0.385 (0.082–1.822)
rs4633			
DOM	1.504 (0.728–3.108)	1.427 (0.611–3.334)	1.523 (0.626–3.707)
REC	2.634 (0.491–14.134)	3.255 (0.522–20.286)	2.418 (0.324–18.037)
rs165655			
DOM	0.902 (0.411–1.979)	0.660 (0.262–1.663)	0.711 (0.276–1.827)
REC	1.020 (0.391–2.662)	0.994 (0.314–3.144)	0.900 (0.262–3.098)
rs165656			
DOM	1.511 (0.728–3.316)	1.418 (0.598–3.362)	1.532 (0.616–3.813)
REC	1.727 (0.394–7.577)	2.118 (0.411–10.906)	1.545 (0.237–10.079)
rs165722			
DOM	1.307 (0.627–2.723)	1.233 (0.525–2.899)	1.320 (0.539–3.237)
REC	2.148 (0.378–12.207)	3.023 (0.465–19.631)	2.214 (0.291–16.882)

^1^: Adjusted for none covariate; ^2^: Adjusted for gender, grade, and sports team membership; ^3^: Adjusted for gender, grade, sports team membership, age, body mass index, waist-hip ratio, screen time, and PA participation; SNPs, single nucleotide polymorphisms; DOM, dominant model; REC, recessive model.

**Table 5:** Haplotype frequencies of the SNPs in the COMT gene in the PARIs cases and the non-PARI controls.

Block	Haplotype	Controls	Cases	*χ^2^*	*p*-Value	OR (95% CI)
1 ^a^						
	C-A-C-C	0.385	0.452	1.137	0.286	1.490 (0.876–2.533)
	C-G-C-G	0.393	0.268	4.333	0.037	0.474 (0.269–0.834)
	T-A-T-C	0.197	0.270	1.854	0.173	1.466 (0.786–2.735)
2 ^b^						
	C-G	0.499	0.522	0.131	0.717	1.111 (0.664–1.860)
	A-A	0.443	0.441	0.001	0.980	0.992 (0.590–1.666)
	A-G	0.059	0.037	0.621	0.431	0.577 (0.164–2.026)

^a^: The bases of block one are listed in the following order: rs165722, rs6269, rs4633, and rs4818; ^b^: The bases of block two are listed in the following order: rs9265 and rs165655; SNPs, single nucleotide polymorphisms; COMT, catechol-O-methyltransferase.

## Data Availability

The datasets used and analyzed in the study are available from the corresponding author on reasonable request.

## Supplementary Materials

The following are available online at https://www.mdpi.com/article/10.3390/ijerph182010828/s1, Table S1. Basic information of primers for the SNPs in COMT gene; Table S2. Basic information of the eight SNPs within COMT gene.
