# Supplementary material for: Association of COMT Polymorphisms with Multiple Physical Activity-Related Injuries among University Students in China
Source: Int J Environ Res Public Health. 2021 Oct 15;18(20):10828. doi: 10.3390/ijerph182010828 (PMC8535648; doi:10.3390/ijerph182010828)
Supplement: Supplementary file 1 [file ijerph-18-10828-s001.zip › ijerph-1397887-supplementary.pdf]

Table S1. Basic information of primers for the SNPs in COMT gene.

SNPs	2nd_PCRP	1st_PCRP	UEP_SEQ
rs9265	ACGTTGGATGAGTAGAGAGCCTTTC TCCAG	ACGTTGGATGACTGCCGAAGGTCA GTCCC	aAGCCCATGCTGGCTG
rs4680	ACGTTGGATGTTTTCCAGGTCTGAC AACGG	ACGTTGGATGACCCAGCGGATGGT GGATTT	GCACACCTTGTCCTTC A
rs6269	ACGTTGGATGTTGCTTGGAGTGCCA CCATC	ACGTTGGATGCAAGGCTGGCATTT CTGAAC	aATCGCCCCCTTGTGTT
rs4818	ACGTTGGATGCAGTCGGGGTTGATC TCGAT	ACGTTGGATGACTGTGGCTACTCA GCTGTG	ttGGTTGATCTCGATGG TGAT
rs4633	ACGTTGGATGTCATGGGTGACACCA AGGAG	ACGTTGGATGTAGGTGTCAATGGC CTCCAG	aatGAGCAGCGCATCCT GAACCA
rs165655	ACGTTGGATGTTCCAAAGCTTCCTT GCCCA	ACGTTGGATGTTCTAAAGGCGGAG GGTCTC	aatgCCTTGCCCAGGGCT G
rs165656	ACGTTGGATGGTGCTTTGAAGGTGA GTTGG	ACGTTGGATGATGTGCTCAGCAGA CCTGTG	ggcaAGGGTGGGTACAG ATTCC
rs165722	ACGTTGGATGTGCTTCCCTGTTCTCT TCTG	ACGTTGGATGAGGCAAGGAGGCA GGCCTA	ccTTCTCTTCTGCTCTGT C

SNPs, single nucleotide polymorphisms; COMT, catechol-O-methyltransferase.

Table S2. Basic information of the eight SNPs within COMT gene.

SNPs	Location [a]	Allele Origin [b]	MAF [c]	*P*-value [d]
rs9265	Chr22:19970108	A/C	0.494	0.355
rs4680	Chr22:19963748	A/G	0.287	0.347
rs6269	Chr22:19962429	G/A	0.323	0.839
rs4818	Chr22:19963684	G/C	0.317	0.544
rs4633	Chr22:19962712	T/C	0.256	0.804
rs165655	Chr22:19970240	A/G	0.457	0.272
rs165656	Chr22:19961340	C/G	0.250	0.621
rs165722	Chr22:19961490	T/C	0.244	0.917

[a]: Location given according to NCBI datasets; [b]: The nucleotide of each SNP shown in left represents the minor allele, as given in NCBI datasets; [c]: Data were calculated from our present dataset; [d]: *P*-value was calculated by χ^2 test for the Hardy-Weinberg equilibrium; SNPs, single nucleotide polymorphisms; COMT, catechol-O-methyltransferase; MAF, minor allele frequency.
